# Study and Characterization of an Ancient European Flint White Maize Rich in Anthocyanins: Millo Corvo from Galicia

**DOI:** 10.1371/journal.pone.0126521

**Published:** 2015-05-11

**Authors:** Chiara Lago, Michela Landoni, Elena Cassani, Enrico Cantaluppi, Enrico Doria, Erik Nielsen, Annamaria Giorgi, Roberto Pilu

**Affiliations:** 1 Dipartimento di Scienze Agrarie e Ambientali—Produzione, Territorio, Agroenergia, Università degli Studi di Milano, Via Celoria 2, 20133, Milano, Italy; 2 Dipartimento di Bioscienze, Università degli Studi di Milano, Via Celoria 26, 20133, Milano, Italy; 3 Dipartimento di Biologia e Biotecnologie, Università degli Studi di Pavia, Via Ferrata 9, 27100, Pavia, Italy; 4 Centre for Applied Studies in the Sustainable Management and Protection of the Mountain Environment—Ge.S.Di.Mont., Università degli Studi di Milano, Brescia, Italy; Chinese Academy of Sciences, CHINA

## Abstract

In the second half of the last century, the American dent hybrids began to be widely grown, leading to the disappearance or marginalization of the less productive traditional varieties. Nowadays the characterization of traditional landraces can help breeders to discover precious alleles that could be useful for modern genetic improvement and allow a correct conservation of these open pollinated varieties (opv_s_). In this work we characterized the ancient coloured cultivar “Millo Corvo” typical of the Spanish region of Galicia. We showed that this cultivar accumulates high amounts of anthocyanins (83.4 mg/100g flour), and by TLC (Thin Layer Chromatography) and HPLC (High Pressure Liquid Chromatography) analysis, we demonstrated that they mainly consisted of cyanidin. Mapping and sequencing data demonstrate that anthocyanin pigmentation is due to the presence of the *red color1 gene*(*r1*), a transcription factor driving the accumulation of this pigment in the aleurone layer. Further chemical analysis showed that the kernels are lacking in carotenoids, as confirmed by genetic study. Finally a DPPH (2,2-diphenyl-1-picrylhydrazyl) radical scavenging ability test showed that Millo Corvo, even though lacking carotenoids, has a high antioxidant ability, and could be considered as a functional food due to the presence of anthocyanins.

## Introduction

The beginning of maize (*Zea mays* ssp. *mays*) domestication has been dated to around 8700 years before the present in Mexico [1–4]. Then the progressive spread of the cultivated crop into the tropical regions and throughout the Americas in the following thousands of years [5–11] allowed hundreds of landraces to adapt and to evolve to suit different environments through human cultivation [12]. After the discovery of the Americas by Europeans, three main maize sources—corn from the American east coast with higher latitude adaptation [13], the photoperiod insensitive CATETO types [13] and the Pearl White [13–14]—played a very important role for the adaptation of maize to Europe. The hybridization of these different corn sources, together with the effects of photoperiod, temperature, humidity and altitude of the different environments allowed the constitution and the differentiation of local European varieties and landraces [13–14]. Hundreds of new landraces have been created in the past 500 years [5, 15]. During this process the farmers’ work of selection, based on specific needs for use and cultivation has been important too: they maintained the landraces as open pollinated populations, creating a collection of corn plants with high heterozygosity and heterogeneity, which represented a very important source of variability and of alleles with high adaptation to the local environments. However in the second half of the last century dent hybrids began to be widely grown in Europe in place of the traditional varieties: these commercial maize cultivars guaranteed superior productivity in response to the need for higher yields [14, 16]. In recent years, renewed interest for the ancient cultivars has been increasing due to the new vision of agricultural systems not only based on yield performance but also on sustainability and the quality of the products.

In this work we characterized an ancient colored landrace, the “Millo Corvo”, cultivated in the Spanish region of Galicia and used to produce a variety of foods. The peculiarity of Millo Corvo is the distinctive dark blue/black coloration of the kernels that confers a typical blue coloration to the bread cooked using this flour.

Maize is able to accumulate pigments in the seeds: carotenoids, that confer the typical yellow to orange color of the seeds and more rarely anthocyanins, conferring a red, purple, blue and black coloration, associated with antioxidant power, thought to be highly beneficial for human health [17].

Carotenoids are hydrophobic C40 isoprenoids that are synthesized in amyloplasts [18]. In maize endosperm those present are mainly lutein and zeaxanthin. In yellow maize there are more than 30 loci involved in the biosynthesis of carotenoids and the main class of mutations that reduce or deplete carotenoids are the *y*_*s*_ conferring white or pale yellow endosperm [19]. In various developing countries white maize is consumed in human diet, even though it is now well understood that Vitamin A, derived from carotenoids, is essential for human health. In fact the World Health Organization estimates that hundreds of millions of persons (in particular children) worldwide suffer from vitamin A deficiency (VAD) [20]. The anthocyanin biosynthetic pathway in maize is known to be controlled by at least two classes of regulatory genes, both of which are required for tissue specific pigmentation of plant and seed tissues [12]. The *R1/B1* family encodes proteins with sequence homology to the basic helix-loop-helix (bHLH) DNA binding domain of the MYC oncoproteins [21], while the *C1/Pl1* family encodes proteins with sequence homology to the DNA-binding domains of the MYB-related oncoproteins [22, 23]; the presence of one member of each family and their interaction allow the activation of the approximately 20 structural gene required for anthocyanin pigment production [24]. In nature many different alleles of these regulatory genes exist, each one driving a tissue-specific coloration [25].

## Materials and Methods

### Plant and sampling material

The Millo Corvo maize variety (from the Spanish region of Galicia), the B73 inbred line (provided by Stock Center Resources of MaizeGDB, http://www.maizegdb.org/stock.php), the Scagliolo variety (from Carenno LC, VA1210) and the Ottofile variety (from Zinasco, PV, VA61) were cultivated in the experimental field of the University of Milan located in Landriano (PV), Italy (N 45°18′, E 9°15′). For all genotypes tested, about 100 seeds were sown in adjacent rows, under the same agronomic conditions. These plants were selfed and the ears obtained were harvested at the same time at the end of the season. About 70 ears of Millo Corvo were shelled and the seeds obtained mixed to create a single bulk used for the determination of anthocyanins, flavonols and phenolic acid. The same was done for the B73 inbred line used as colorless control. For the anti radical power (ARP) determination we used the Millo Corvo seeds bulk described above, a Scagliolo seeds bulk obtained in the same way and the segregant yy seeds (without carotenoids) obtained by selfing the progeny Millo Corvo x B73.

### Milling

Flour samples were obtained using a ball mill (Retsch MM200, Retsch GmbH Germany), and seeds (cleaned from the glumes) were ground for 5 min at 21 oscillations s^−1^ frequency.

### Spectrophotometer determination of anthocyanins, flavonols and phenolic acids

Five mg of flour was first boiled with 100 μl of distilled water for 30 minutes and then left in an overnight agitation with 1 ml of the extraction buffer (1% HCl, 95% ethanol). After another agitation time of 2 hours with 500 μl of extraction buffer, the supernatants were collected together and centrifuged for 30 minutes. Their absorbance was determined spectrophotometrically at 530 nm for anthocyanins, at 350 nm for flavonols and at 280 nm for phenolic acids [26].

The amount of anthocyanins was calculated as cyanidin 3-glucoside equivalents (molar extinction coefficient (ɛ) 26900 Lm^-1^ mol^-1^, M.W. 484.82), flavonols content as quercetin 3-glucoside equivalents (ɛ 21877 Lm^-1^ mol^-1^, M. W. 464.38) and the amount of phenolics as ferulic acid equivalents (ɛ 14700 Lm^-1^ mol^-1^, M.W. 194.18). The analyses were conducted four times for each genotype, and the confidence interval (C.I.) at 95% was calculated.

### Qualitative determination of anthocyanins: TLC (Thin Layer Chromatography) and HPLC (High Performance Liquid Chromatography)

The fine powder of the pericarp and aleurone layers of the Millo Corvo kernels (obtained using a manual electric drill) was boiled at 100°C in 2 ml of 2N HCl for 40 minutes. After adding 1 ml of isoamyl alcohol, the upper phase was dried and suspended in EtOH 95% and HCl 1% for the TLC analysis and in methanol for the HPLC run. For TLC analysis, cyanidin, pelargonidin and delphinidin standards were loaded together with the extracts on a pre-coated plastic sheet (Polygram Cel 300, Macherey-Nagel) for TLC using formic acid: HCl: water 5:2:3 as solvent. Developed plates were dried and pictured with a digital camera (A430 Canon) using both white and UV illumination.

For HPLC 20 μl of the sample were injected in an HPLC Kontron Instrument 420 system equipped with a C18 column Zorbax ODS column, 250 mm X 4.6 mm, 5 μm, Teknokroma (Agilent Technologies, Santa Clara, CA, USA) and the absorbance at 530 nm was monitored. Anthocyanins quantification was performed by the method used by Astadi [27]; the HPLC conditions were as follows: from min 0 to 8 min, solvent A (10% formic acid) from 96 to 85%, solvent B (100% Acetonitrile) from 4 to 15%; from min 8 to 25, solvent B was kept at 15%; from min 25 to 27, solvent A 20%, solvent B 80%; from min 27 to 30, solvent A 80%, solvent B 20%. The flow rate was 1 ml/min.

### Qualitative determination of seed carotenoids: HPLC

After incubating 1 g of maize flour in 3 ml of hexane/acetone 1:1 solution with 100 mg/ml of BHT for 30 min at room temperature, the sample was dried by means of a speedvac and the pellet was dissolved in 3 ml of hexane and washed three times with 4 ml of distilled water in order to remove the hydrophilic compounds. Sample extracts were concentrated by speedvac and immediately analysed.

Carotenoids were assayed by an HPLC method adapted from that described by Tukaj et al. [28] using a Kontron Instrument 420 system, equipped with C18 reverse-phase Zorbax ODS column, 250 9 4.6 mm, 5 lm (Agilent

Technologies, Santa Clara, CA, USA). The solvent initially consisted of 60% solvent A (methanol—ammonium acetate 80/20 v/v) and 40% solvent B (methanol/acetone, 80/20 v/v), which finally was brought to 0% solvent A and 100% solvent B over a period of 20 min and fluxed under these conditions for 5 additional minutes. The column was subsequently returned to its original mobile phase (60% solvent A and 40% solvent B) over the next 5 minutes, and fluxed under these conditions for 5 additional minutes prior to the injection of a new sample. The solvent flow rate was 1 ml min^–1^.

### Mapping

Millo Corvo was mapped in segregating F2 populations using the *bnlg1028* simple sequence repeat (SSR) marker chosen on chromosome 10 (bin 10.06) from MaizeGDB (http://www.maizegdb.org). A total of 85 F2 seeds (obtained by selfing the progeny of the cross B73 x Millo Corvo) were screened for color and each flour was used for DNA extraction [29]. Polymerase chain reactions were performed in a final volume of 10 μl and the reactions were carried out as follows: 94°C for 2 min, 35 cycles at 94°C for 1 min, 57°C for 1 min, 72°C for 1 min, and a final step at 72°C for 5 min. The amplified fragments were resolved on 3% agarose gels. Recombinant values were converted to map distance using MAPMAKER3 [30].

### Histological analysis of Millo Corvo seeds

For light microscopy studies, coloured Millo Corvo and colourless B73 seeds were imbibed in water overnight and fixed in freshly prepared 4% paraformaldehyde (Sigma P4168) in PBS (130mM NaCl, 7mM Na_2_HPO_4_, 3mM NaH_2_PO_4_.H_2_O) at 4°C overnight, then rinsed in 0.85% NaCl and transferred in 70% ethanol at 4°C until processed. Following successive dehydration in ethanol series and embedding in Paraplast Plus (Sigma P3683), 15μm-thick sections were cut and serially arranged on microscope slides. To preserve anthocyanin pigments *in situ*, sections were mounted on slides using tert-butyl alcohol instead of water. Images were taken using a Zeiss IMAGE R.D1 microscope equipped with an AxioCam MRc1 camera.

### Amplification and sequencing

The presence of the *R-g* allele involved in the coloration of the Millo Corvo kernels has been determined by sequencing: genomic DNA was amplified by high fidelity PCR (Pfu polymerase; Stratagene, La Jolla, CA, USA) using the specific primers OR31 (5'-ATGGCTTCATGGGGCTTAGATAC-3') and OR32 (5'-GAATGCAACCAAACACCTTATGCC-3') for *R1* gene [31]. Four sequences coming from independent amplification were sequenced in outsourcing. To deduce the consensus DNA sequence we used the freely available computer software CLUSTALW (http://www.ebi.ac.uk/clustalw/). To study the sequence obtained we used BLAST (http://www.ncbi.nlm.nih.gov/BLAST/).

### Anti Radical Power (ARP) determination

The antioxidant ability of the pigments was inferred by the comparison of the Anti Radical Power (ARP) possessed by the white and colored kernels of a Millo Corvo segregating synthetic population, using the DPPH (2,2-diphenyl-1-picrylhydrazyl) free radical-scavenging activity method [32].

Acetone 70% (acetone:water 70:30 v/v) was added to an aliquot of the fine powder, keeping the ratio 1:4 (w/v). The mixture was shaken at 4°C in the dark for 3 hours, then centrifuged to collect the clean extracts. A 0.12 mM ethanolic DPPH solution was added to increasing aliquots of each sample and the final volume adjusted to 2.50 ml. The absorbance of the discolorations of the DPPH in ethanol and of the samples were measured at 516 nm after incubation for 2 hours at room temperature in the dark, until the reaction reached the steady state.

The percentage of scavenged DPPH values was calculated and then plotted against the extract volumes so as to calculate by interpolation the amount of extract required to consume 50% of the initial DPPH amount [33]. The ARP is the reciprocal of this value [34]. The analyses were conducted three times for each genotype.

## Results

### Phenotypic characterization of the Millo Corvo landrace

The Millo Corvo traditional open pollinated variety was cultivated in the field at Landriano (PV) from April to September, during this period some agronomic traits were measured (Table 1). The plants reached maturity in about 90 days after sowing in this environment. The plants were, on average, 248.36 cm in height, with the ears positioned at 105.09 cm from the soil. The ears were of cylindrical-conical shape with 12 rows, measuring 16.26 cm in length with a cob diameter of 2.75 cm (Table 1). The kernels were flint type and pigmented, with an average weight of 0.319 g (Table 1); each ear weighed about 109.26 g for an estimated yield of about 6–7 tons per hectare (sowing 6–7 seeds per square meter). As control Ottofile and Scagliolo varieties, out of more than 700 catalogued open pollinated traditional Italian flint maize, were cultivated and measured in the same conditions (Table 1).

**Table 1:** Agronomic parameters of Millo Corvo, Ottofile and Scagliolo cultivars cultivated at Landriano (PV).

Parameters	Millo Corvo	Ottofile	Scagliolo
**Plant height (cm)**	248.36 ± 10.90	198.17 ± 12.30	172 ± 16.30
**Ear height (cm)**	105.08 ± 6.04	112.35 ± 8.30	64 ± 5.30
**Ear length (cm)**	16.26 ± 0.77	21.49 ± 1.24	16.35 ± 1.38
**Cob diameter (cm)**	2.75 ± 0.21	1.93 ± 0.28	2.23 ± 0.27
**Kernels weight per ear (g)**	109.25 ± 26.02	54.14 ± 13.74	99.19 ± 17.66
**Seed weight (g)**	0.31 ± 0.02	0.26 ± 0.08	0.24 ± 0.12
**No. of rows**	12	8	12

Confidence Intervals at 95% are shown, n > 50.

### Characterization of seed pigment: anthocyanins, flavonols and phenolic acids

**Fig 1:**
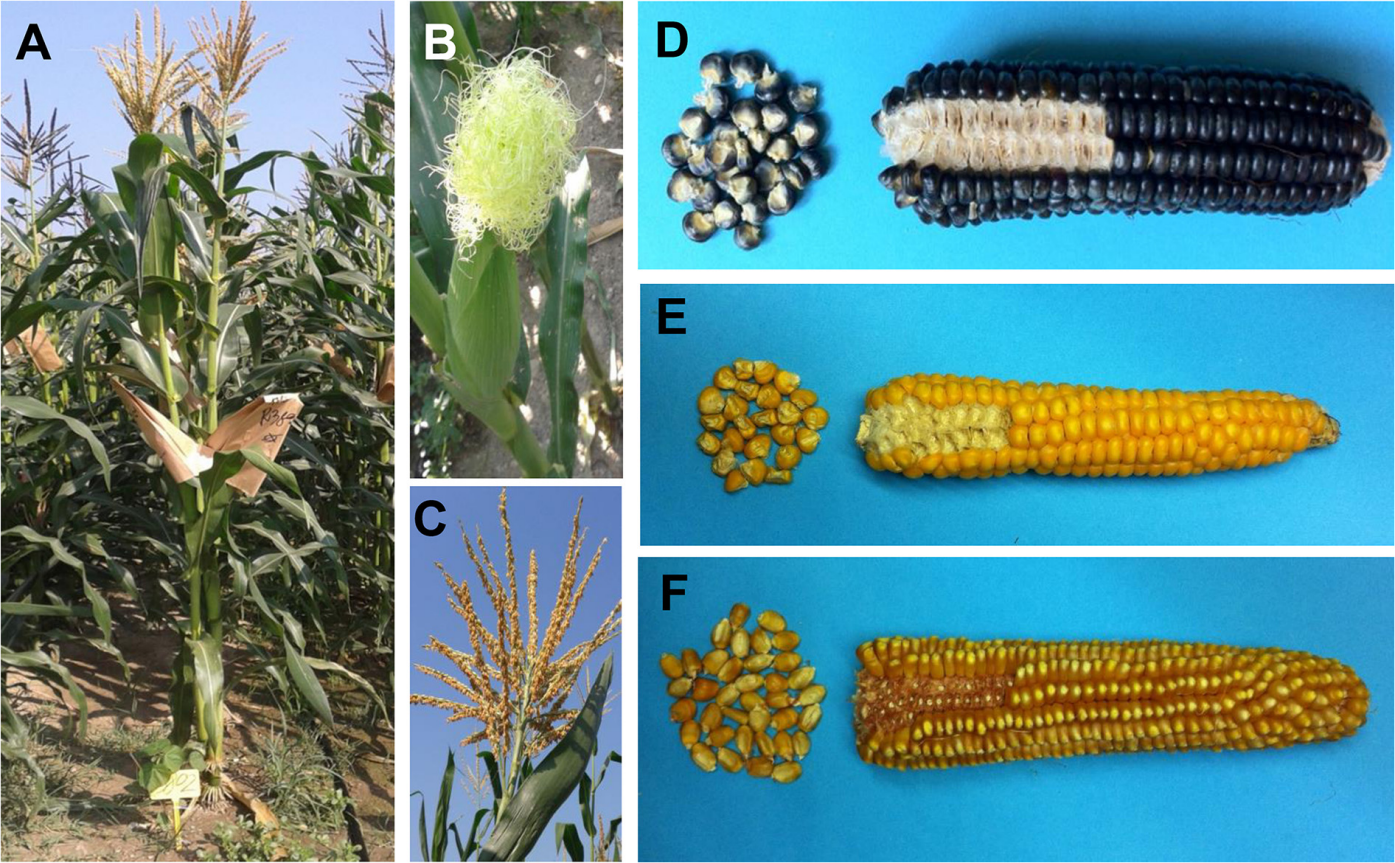
Phenotype of the Millo Corvo maize cultivar and ears comparison with two other maize traditional cultivars. (A) Plant at maturity, (B) immature ear with silks, (C) tassel and (D) ear of Millo Corvo cultivar, (E) ear of Ottofile cultivar and (F) ear of Scagliolo cultivar.

**Fig 2:**
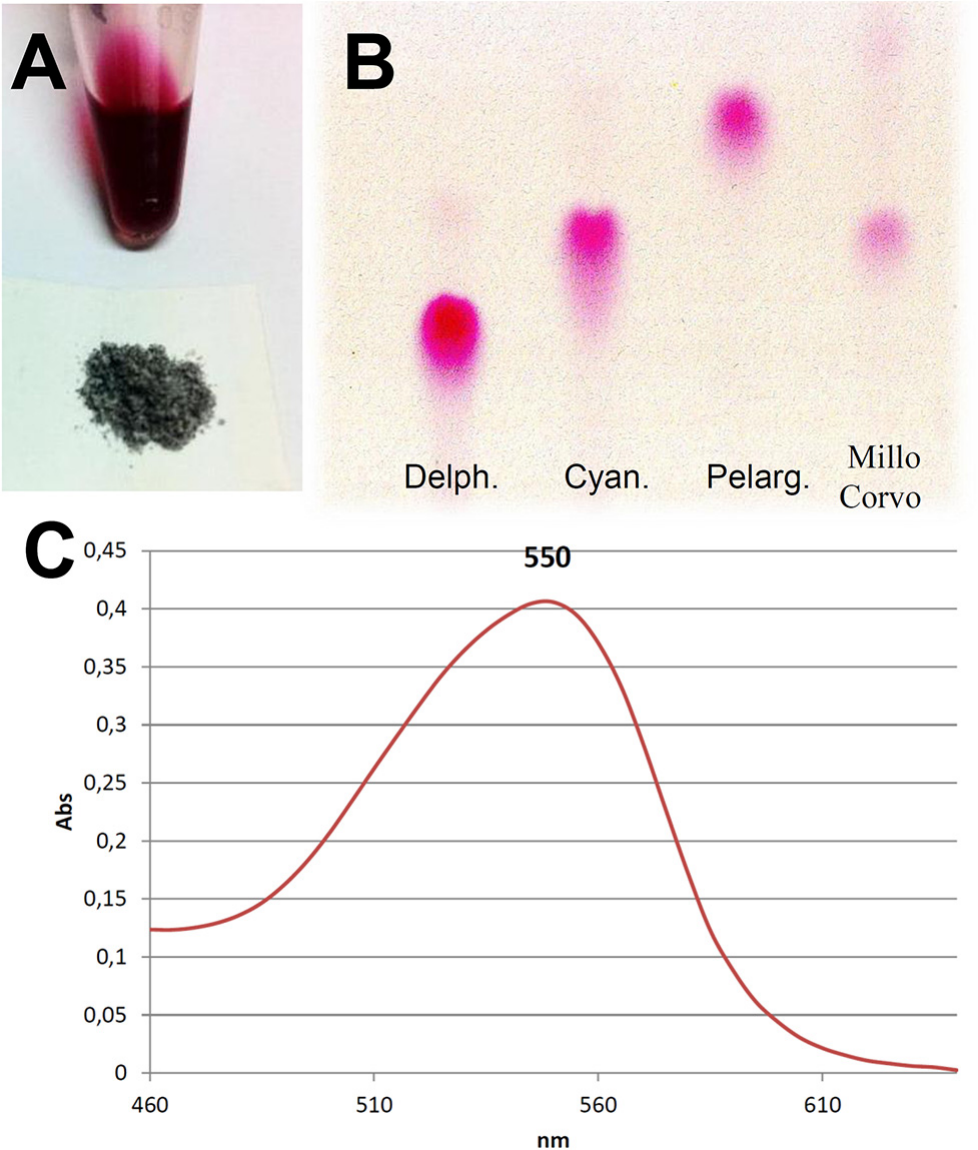
Anthocyanin characterization. Anthocyanins alcoholic extract from the powder (A, above) obtained by milling the surface of the Millo Corvo kernels (A, below). TLC analysis (B) and absorbance spectrum of the extract (C). The standard used for the TLC analysis were: cyanidin (cyan.), delphinidin (delph.) and pelargonidin (pelarg.).

**Fig 3:**
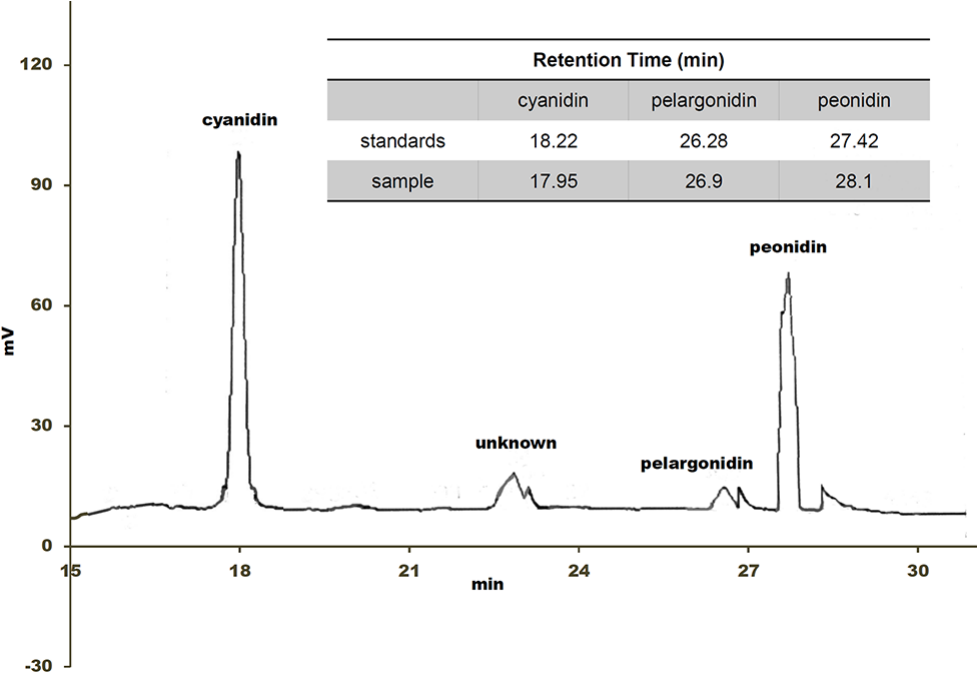
HPLC analysis. HPLC chromatogram of the anthocyanins extracted from the Millo Corvo seeds and the corresponding retention times compared to the standards cyanidin, pelargonidin and peonidin.

The important peculiarity of this variety is surely the seed color, that would seem to be the only pigmented tissue with the exception of the seedling (Fig 1 and S1 Fig). It is well known that maize plants can accumulate anthocyanins and for this reason we conjectured that the pigments observed in the Millo Corvo seeds were flavonoids and in particular anthocyanins. Table 2 shows the spectrophotometric results on the amounts of anthocyanins, flavonols and phenolic acids present in the seed flour of the Millo Corvo, in comparison to the colorless B73 inbred line: we found respectively 83.45 mg/100g of flours, 74.21 mg/100g, 216.63 mg/100g in the Millo Corvo variety, while 3 mg/100g, 66 mg/100g, 113 mg/100g in the B73 line. We used the B73 inbred line as representative of all the classical yellow maize cultivars where the pigments are not present or present as trace. Anthocyanins are a very wide group of pigments, so to better characterize them, we performed Thin Layer Chromatography (TLC) and High Performance Liquid Chromatography (HPLC). We did not analyze from a qualitative point of view the colorless controls (B73, Scagliolo and Ottofile) because the very low amount of anthocyanins as shown for the classical yellow inbred line B73. In Fig 2B the TLC plate shows that the main spot present in the Millo Corvo extract is due to the cyanidin molecule: according to the standards loaded in the plate and considering that the absorption peak of the anthocyanins extract at 550 nm is close to the typical peak of cyanidin at 545 nm (Fig 2C). Another little spot, poorly visible and not identified, has been detected with a run length higher than those of the standards (Fig 2B). The following HPLC analysis (Fig 3) confirmed that cyanidin is the most abundant anthocyanin in Millo Corvo, representing 65.90% of the total anthocyanidin molecules; this analysis also detected 31.40% of peonidin, 1.96% of pelargonidin (S2 Fig).

**Table 2:** Spectrophotometric quantification of anthocyanins, flavonols and phenolic acids, quantified as mg cyanidin-3-glucoside equivalents, quercetin 3-glucoside equivalents and ferulic acid equivalents respectively per 100 g of dry seed flour.

	Anthocyanins (mg/100g)	Flavonols (mg/100g)	Phenolic Acids (mg/100g)
**Millo Corvo**	83.45 ± 11.44	74.21 ± 17.83	216.63 ± 29.05
**B73**	3 ± 1	66 ± 10	113 ± 0.2

The analyses were conducted four times for each genotype, and the confidence interval at 95% was calculated.

### Characterization of seed pigment: carotenoids

**Fig 4:**
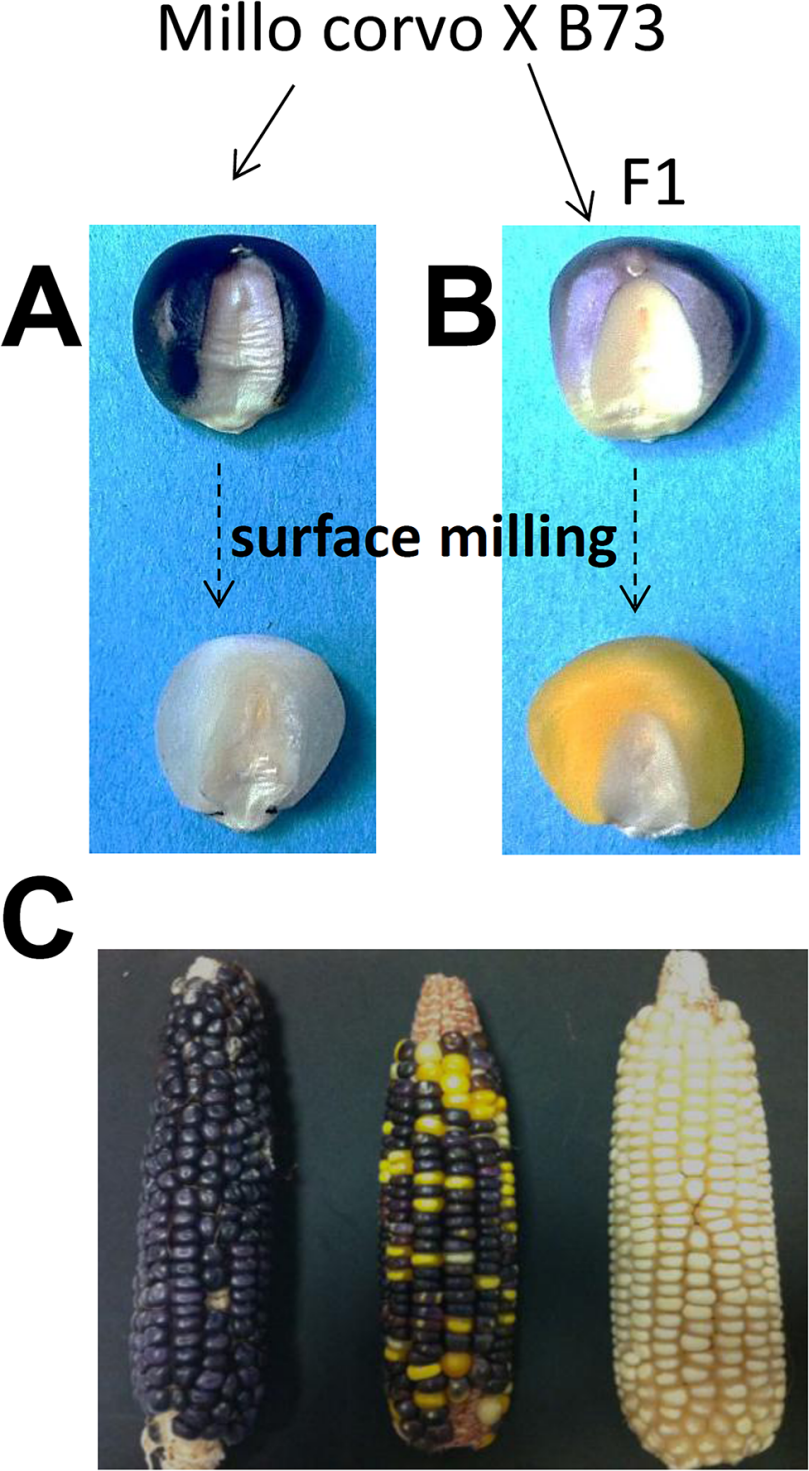
Carotenoid assay by surface milling and following F2 and F3 *yy* segregation. Surface milling of the Millo Corvo seeds (A) and of the F1 seeds obtained by crossing with B73 inbred line (B). In (C) from left to right we can see Millo Corvo ear, F2 segregating ear and a F3 ear obtained selecting and sowing F2 seeds without carotenoids.

Unexpectedly, in the Millo Corvo seeds’ flour, the HPLC analysis found that the amount of carotenoids present was under the detectable threshold. We hypothesized that the Millo Corvo cultivar carried a recessive homozygous mutation belonging to the white endosperm class (*y*_*s*_) whose phenotype effect was hidden because of the anthocyanins accumulation. To confirm this hypothesis we used a hand drill to mill the seeds’ surface, where the anthocyanins were accumulated: as expected we demonstrated the absence of carotenoid in the inner layer of the seed, which appeared completely white (Fig 4A) as reported for the *y*_*s*_ recessive mutation [35]. Furthermore, as expected for a recessive mutation, crossing the Millo Corvo with the B73 line (able to produce carotenoids) we obtained yellow F1 seeds after surface milling (Fig 4B). These results were further strengthened by studying the F2 segregating progeny for the *yy* seeds and the following F3 ears obtained selecting and sowing the *yy* seeds (Fig 4C).

### Genetic constitution and heritability of the colored seed trait

It is well known that anthocyanins can be accumulated in the pericarp layer, a tissue of maternal origin, or in the aleurone, the outer layer of the seed endosperm [24].

**Fig 5:**
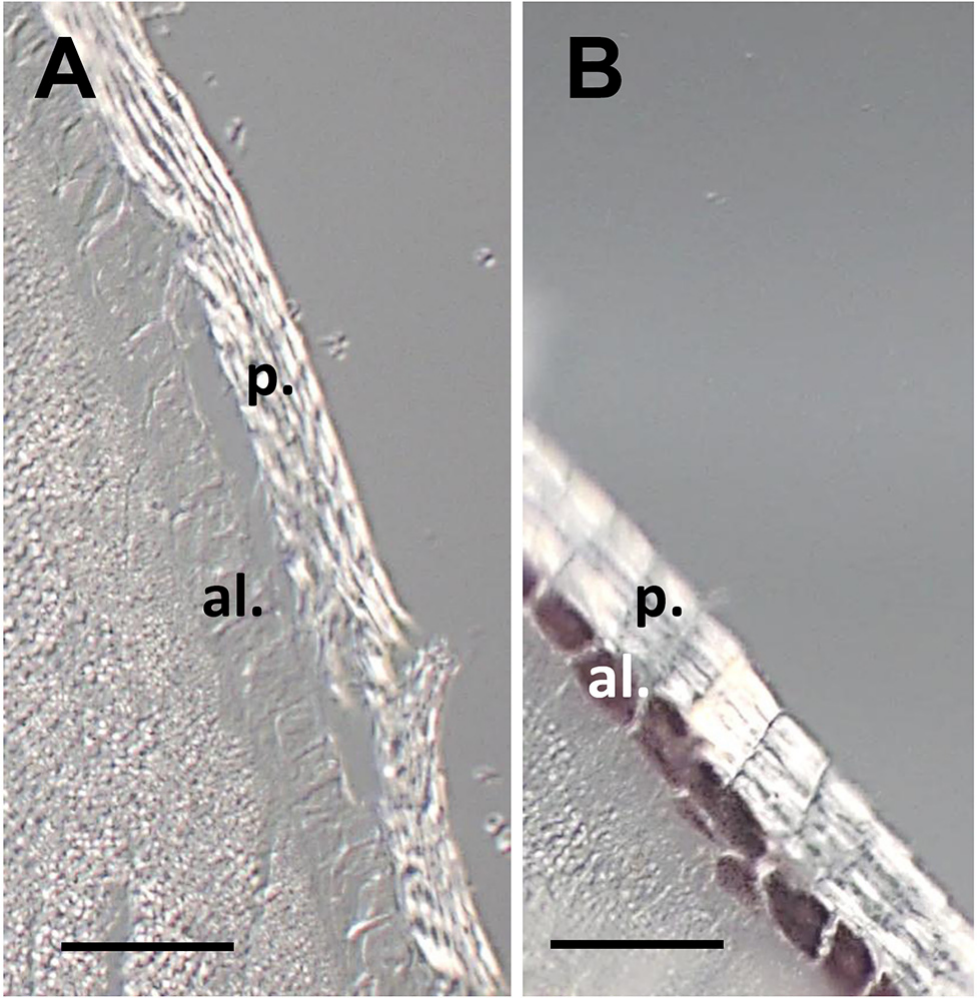
Histological analyses of seeds preserving anthocyanin pigments in situ. (A) B73 colourless seed used as control and (B) Millo Corvo seed. al. aleurone layer; p. pericarp layer. Bar = 100 μm.

With the aim to identify the tissues where anthocyanins were accumulated and to understand the heritability of the trait “seed pigmentation” in the Millo Corvo variety we studied the progeny of an F2 population obtained as described in the previous paragraph. As shown in Fig 4, the pigmentation of the F1 seeds (obtained using the B73 plant as female and Millo Corvo as pollen donor) was weak compared to the Millo Corvo. This finding suggested a dosage effect typical of pigments accumulated in aleurone layers: in Millo Corvo aleurone, three doses of genes involved in the pigmentation (aleurone is a triploid tissue) seemed to be present whilst in the F1 there was only one. Furthermore the presence of the pigment just in F1 seeds excluded the possibility that it was a pericarp pigmentation that would appear in the next generation (being a tissue of maternal origin). Selfing F1 plants we obtained an F2 progeny segregating 3:1 for seed color (S3 Fig), confirming that the pigmentation is under the control of a monogenic dominant character that drives the accumulation of anthocyanins (mainly cyanidin) in the aleurone layer. The genetic data were confirmed by histological analysis of transverse sections of mature seeds showing the pigmentation only in the aleurone layer (Fig 5). This evidence led us to think that the regulatory *r1* gene may be responsible for the seed anthocyanin biosynthesis. To strengthen this finding we mapped the character “seed pigmentation” using SSR markers to confirm the presence of the “colored seed” trait on the long arm of chromosome ten where *r1* locus maps (bin 10.06). We used genomic DNA obtained by F2 mapping populations of 85 F2 seeds screened for color and genotyped using the *bnlg1028* simple sequence repeat (SSR) marker mapping on chromosome 10 (bin 10.06). We found an association of 3.4 cM between the trait “seed color” and *bnlg1028*.

### Molecular analysis of the *r1* gene

*R1* gene is a complex locus composed of two distinct components: the *S1* and *S2* component driving the pigmentation of the seed and the *P* component driving the pigmentation of the plant tissues [36]. When an allele at the *r1* locus carries both the components it is named *R-r*. If intrachromosomal rearrangement occurs (typical in complex genes) and the allele loses *S* components, *r-r* alleles are formed; when the *P* component is lost, alleles of class *R-g* are formed, and when both are lost we have *r-g* alleles unable to confer any plant pigmentation [25, 36, 37]. In the case of Millo Corvo the pattern of pigmentation of the *R1* gene is similar to that of *R-g* (Table 3) in fact in our case we have the seed colored and the plant colorless with the exception of the seedling (S1 Fig).

**Table 3:** Tissue specific expression of the main classes of *r1* alleles.

Allele	Pigmentation	
	Seed	Plant
***R-R***	+	+
***R-g***	+	-
***r-r***	-	+
***r-g***	-	-

To confirm these data we sequenced a 3' portion of *R1* gene using specific primers (see Material and Methods chapter). The sequencing of 4 independent amplicons and the following alignment with the CLUSTALW program allowed us to obtain a consensus sequence of 454 nucleotides (GenBank accession number: BankIt1769632 Seq1 KP056782) used for the research by the BLASTN program. The results obtained confirmed the presence of an *R-g* allele in the Millo Corvo cultivar, in fact we found significant alignments with the sequence NM_001112603.1, the seed color component at *R1* (*S*) mRNA of *Zea mays* (S4 Fig).

### Antioxidant ability of the Millo Corvo flour

**Fig 6:**
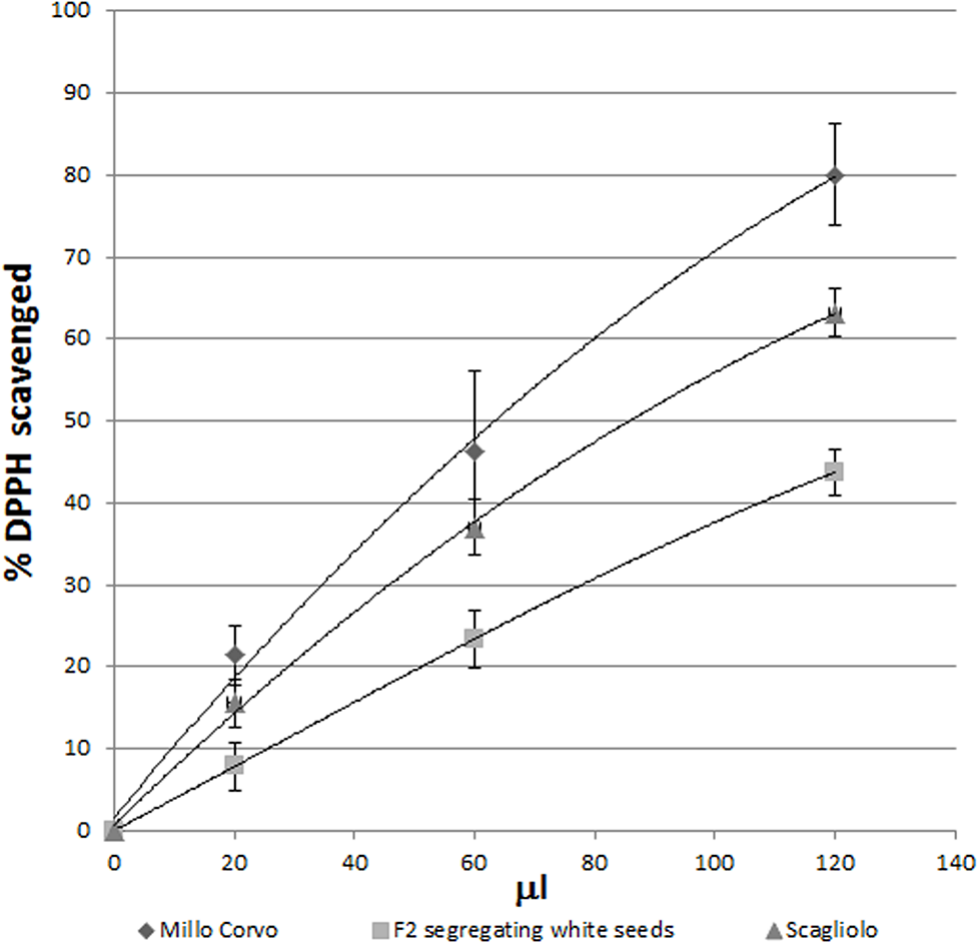
DPPH radical scavenging test. Comparison of the antioxidant ability in the DPPH radical scavenging test of Millo Corvo (accumulating anthocyanins and no carotenoids), F2 segregating white seed (lacking anthocyanins and carotenoids) and Scagliolo cultivar (accumulating carotenoids) flours. Error bars represent SD (n = 3).

To detect the antioxidant ability conferred by the anthocyanin molecules, a DPPH assay was performed on the flour obtained from the Millo Corvo seeds (containing anthocyanins but not carotenoids) and from F2 white segregating seeds (without anthocyanins and carotenoids). We also analyzed as control the yellow Scagliolo variety, a popular Italian polenta variety (containing only carotenoids). The percentage of scavenged DPPH values were calculated and then plotted against the extracted volumes (Fig 6). The antioxidant results were expressed as Anti Radical Power (ARP) as suggested by Doria and colleagues [34]. The colored Millo Corvo seeds showed the highest antioxidant ability with 0.06 of ARP, the Scagliolo variety had a value of 0.04, while the F2 white seeds showed the lowest antioxidant power with 0.03 as expected, since they lack carotenoids and anthocyanins.

## Discussion

Starting from the last century, the increased needs for corn have focused the farmers’ attention on the dent hybrids with high yields, displacing the maize landraces with the risk of losing their sources of genetic variability. Now the institutions and the companies responsible for conducting maize genetic improvement are starting to study the ancient landraces across the continents with the aim of identifying and using novel alleles and haplotypes in a context of low input and sustainable agriculture [38]. In this scenario the study of the immense maize genetic diversity present around the world has a big limiting factor in the requirement for conscious protection of open pollinated varieties and their precise characterization.

For these reasons the Millo Corvo ancient landrace from the Galician Spanish region has been studied and characterized. The most obvious characteristic of this cultivar is the blue/black pigmentation of the seed (Fig 1D) which differentiates it for example from Ottofile (Fig 1E) and Scagliolo (Fig 1F) traditional Italian cultivars, which as well as most other maize varieties are not able to produce flavonoid pigments. It is well known that red/black coloration of maize kernels is due to the accumulation of flavonoids and in particular anthocyanins [39] and with the aim to quantify this pigment a spectrophotometric quantification of the main class of molecules was performed. As reported in Table 3, significant differences were found, as expected, for anthocyanins and phenolic acids amounts whilst no difference was noted for the flavonols content in comparison to the well characterized B73 inbred line used as typical control of all the colorless varieties. These data are in agreement with the work of Lopez-Martinez and colleagues who found a range between 76 and 869 mg/100g of anthocyanins in 18 colored landraces of Mexican maize [40]. With the aim to characterize the anthocyanins present we carried out TLC and HPLC analysis of the pigment. The TLC plate indicated the presence of the cyanidin molecule (Fig 2), confirmed by the HPLC analysis (Fig 3 and S2 Fig). The second spot, poorly visibly in the TLC plate, is probably due to the presence of peonidin, quantified as 31.4% by the HPLC analysis (S2 Fig). Several reports have shown that cyanidin, pelargonidin, and peonidin glycosides are the main anthocyanins present in maize kernels [39, 41, 42], among which cyanidin 3-glucoside is the most abundant one in the dark red, dark blue, light blue and multicolor maize kernels [39]. The presence of anthocyanins in cereals is generally associated with a stronger antioxidant activity and the higher amounts of these phenolic compounds seem to directly contribute to higher antioxidant power [39–40]. The presence of these molecules in the diet is important in the prevention of chronic diseases such as cardiovascular disease, cancers, respiratory disease, diabetes and obesity as shown in numerous papers (reviewed by Tsuda) [42].

Considering that the yellow and white corn varieties do not accumulate anthocyanins in the kernel or only in trace amounts, we can consider the Millo Corvo cultivar a proper functional food. Furthermore the high percentage of cyanidin present (about 66%) in this variety (S2 Fig) represents an important feature because several papers reported the specific beneficial effect of the cyanidin in the diet of animal models. In particular a work by Toufektsian et al. in 2008 reported that chronic dietary intake of a synthetic maize population rich in cyanidin (about the same quantitative present in the Millo Corvo) protected the rat heart against ischemia-reperfusion injury [43].

In this work we also characterized in detail the genetic basis of pigment accumulation showing that the trait “seed colored” is a monogenic dominant character (S3 Fig). Furthermore histological analysis conducted preserving the pigment present in the fresh tissue showed that the pigment is accumulated in the aleurone layer (Fig 5). Taken together the results obtained suggested that in this cultivar there was present an allele of the *R1* regulatory gene of anthocyanin biosynthesis, because typically the *r1 and c1* genes control the aleurone seed pigmentation whilst *b1 pl1* genes control the vegetative tissue (in the seed the pericarp layer is of maternal origin). Further a strong evidence to support our hypothesis was given by the mapping: we demonstrated that the “colored seed” trait maps on the long arm of chromosome 10, where the *r1* gene maps. Further investigations were made to assess which kind of *r1* allele was present in Millo Corvo variety. Comparing the data obtained from tissue specificity pigmentation of Millo Corvo (S1 Fig) with the data on the four principal classes of *r1* alleles (Table 3) we inferred the presence of an allele of *R-g* class. The *r1* gene is a complex locus, made up of three components *P*, *S1*, *S2* which arose by gene duplication [37]. This complex locus undergoes with high frequencies (overall frequency of 6.2 x 10^–4^) genetic rearrangement by intrachromosomal recombination between P and S units, which results in the loss of one *R-r* component and generates the big genetic variability present at this locus [44]. The allele in which all these three components are functional is called *R-r*, *R-g* if the P component is missing, *r-r* if both the S components are missing and *r-g* if all the three components are missing. As we reported, each of these alleles has a specific tissue specificity for the synthesis of the anthocyanins, and given the phenotypic data acquired on Millo Corvo plants in the field and the histological analysis, it can be supposed that the anthocyanin biosynthesis in Millo Corvo is regulated by an *R-g* allele type. This hypothesis has been further confirmed by the sequencing and the following alignment analysis by BLAST program (S4 Fig). Further work will be necessary to better characterize this new allele at the *r1* locus from a molecular point of view, also because of its capacity to accumulate pigment in the seedling tissue that usually is not pigmented in the presence of *R-g* alleles.

Another important source of hydrophobic dietary antioxidants and pigment in maize are carotenoids. Generally carotenoids, and in particular lutein and zeaxanthin, are present in maize varieties with yellow to orange coloration [39, 45]. For example, Berardo et al. [46] found in average around 42.07 mg/kg of total carotenoids in an Italian polenta corn collection; among them, however, there were a few white varieties in which carotenoids were not synthesized. On the other hand in many developing countries around the world the utilization of white maize landraces is widespread, for reasons that so far are not well understood. In fact an adequate daily consumption of carotenoids is essential for human health: its deficiency may cause blindness, increased infectious morbidity and mortality, growth retardation, and anemia [39, 47], as already experienced in Africa where white corn is the main staple food [39, 48]. Our HPLC analysis showed that the Millo Corvo cultivar lacks carotenoids and that this character is controlled by a monogenic recessive mutation, as shown by the study of F1, F2 and F3 progenies (Fig 4). White endosperm is an ancient trait shared with teosinte, the wild progenitor of maize, caused by *ys* recessive mutations impairing carotenoids biosynthesis [49]. It seems likely that Pyrenean-Galician landraces have been developed through hybridization with the Northern US flints introduced into Europe in the sixteenth century from the north of France [5, 15, 50] and we can conjecture that this last parental contribution brought the *y* allele which has been fixed in the following generations. To further characterize the Millo Corvo variety we measured the antioxidant ability of its flour containing anthocyanins and lacking carotenoids in comparison with a Scagliolo cultivar used as control (an Italian polenta maize variety containing carotenoids) and compared the data obtained with an F2 segregating white seed lacking both anthocyanins and carotenoids (Fig 6). The results obtained showed the highest ARP value (0.06) in the dark blue kernels of Millo Corvo and the lowest ARP value (0.03) in the white kernels while the yellow–orange cv. Scagliolo showed an intermediate ARP value of 0.04. These data showed that Millo Corvo even though lacking carotenoid has a higher antioxidant ability, due to the presence of anthocyanins, compared with a classical yellow orange cultivar such as Italian Scagliolo polenta maize. To conclude, this ancient cultivar represents an historic landrace that could be a useful tool in future breeding programs and a promise for the development of functional foods or natural colorants.

## Supporting Information

S1 Fig Tissues in which pigments are accumulated in the Millo Corvo cultivar. (DOCX)

S2 Fig Partition of the anthocyanidins present in the extracts of the Millo Corvo kernels, according to the HPLC analysis. (DOCX)

S3 Fig Segregation of the “seed color” trait observed in the F_2_ progeny obtained by selfing Millo Corvo x B73 plants. The expected segregation values for color trait was 3:1 in the case of the presence of a single dominant gene driving the pigmentation.
(DOCX)

S4 Fig Partial sequencing analysis of *r1* Millo Corvo allele. Alignment obtained by BLASTN program using as query the consensus sequence of 454 nucleotide at the 3' portion of r1 gene.
(TIF)
