# Correction: Study and Characterization of an Ancient European Flint White Maize Rich in Anthocyanins: Millo Corvo from Galicia

**DOI:** 10.1371/journal.pone.0130110

**Published:** 2015-06-03

**Authors:** Chiara Lago, Michela Landoni, Elena Cassani, Enrico Cantaluppi, Enrico Doria, Erik Nielsen, Annamaria Giorgi, Roberto Pilu

The affiliation for the fifth author is incorrect. Enrico Doria is not affiliated with #3 but with #5 CSL (Centre of Sustainable Livelihood), Vaal University of Technology, Vanderbijlpark (South Africa).
