# Supplementary material for: Study and Characterization of an Ancient European Flint White Maize Rich in Anthocyanins: Millo Corvo from Galicia
Source: PLoS One. 2015 May 11;10(5):e0126521. doi: 10.1371/journal.pone.0126521 (PMC4427395; doi:10.1371/journal.pone.0126521)
Supplement: S1 Fig — (DOCX) [file pone.0126521.s001.docx]

| SI1. Tissues in which pigments are accumulated in the Millo Corvo cultivar. | |
| --- | --- |
| Seedling | + |
| Roots | - |
| Stem | - |
| Anthers | - |
| Silks | - |
| Husks | - |
| Cob | - |
| Seed | + |
