# Supplementary material for: Study and Characterization of an Ancient European Flint White Maize Rich in Anthocyanins: Millo Corvo from Galicia
Source: PLoS One. 2015 May 11;10(5):e0126521. doi: 10.1371/journal.pone.0126521 (PMC4427395; doi:10.1371/journal.pone.0126521)
Supplement: S2 Fig — (DOCX) [file pone.0126521.s002.docx]

| SI2. Partition of the anthocyanidins present in the extracts of the Millo Corvo kernels , according to the HPLC analysis. | |
| --- | --- |
| Anthocyanidin | **%** |
| Cyanidin | 65.90 |
| Peonidin | 31.40 |
| Pelargonidin | 1.95 |
