# Supplementary material for: Study and Characterization of an Ancient European Flint White Maize Rich in Anthocyanins: Millo Corvo from Galicia
Source: PLoS One. 2015 May 11;10(5):e0126521. doi: 10.1371/journal.pone.0126521 (PMC4427395; doi:10.1371/journal.pone.0126521)
Supplement: S3 Fig — The expected segregation values for color trait was 3:1 in the case of the presence of a single dominant gene driving the pigmentation. (DOCX) [file pone.0126521.s003.docx]

| SI3. Segregation of the “seed color” trait observed in the F_2_ progeny obtained by selfing Millo Corvo x B73 plants. The expected segregation values for color trait was 3:1 in the case of the presence of a single dominant gene driving the pigmentation. | | | | |
| --- | --- | --- | --- | --- |
|  | **segregation** | | |  |
| cross | **colored** | **colorless** | χ2 value | p |
| (Millo Corvo x B73) selfed | 460 | 171 | 1.48 | 0.3-0.2 |
