# Supplementary figures and images for: Study and Characterization of an Ancient European Flint White Maize Rich in Anthocyanins: Millo Corvo from Galicia
Source: PLoS One. 2015 May 11;10(5):e0126521. doi: 10.1371/journal.pone.0126521 (PMC4427395; doi:10.1371/journal.pone.0126521)

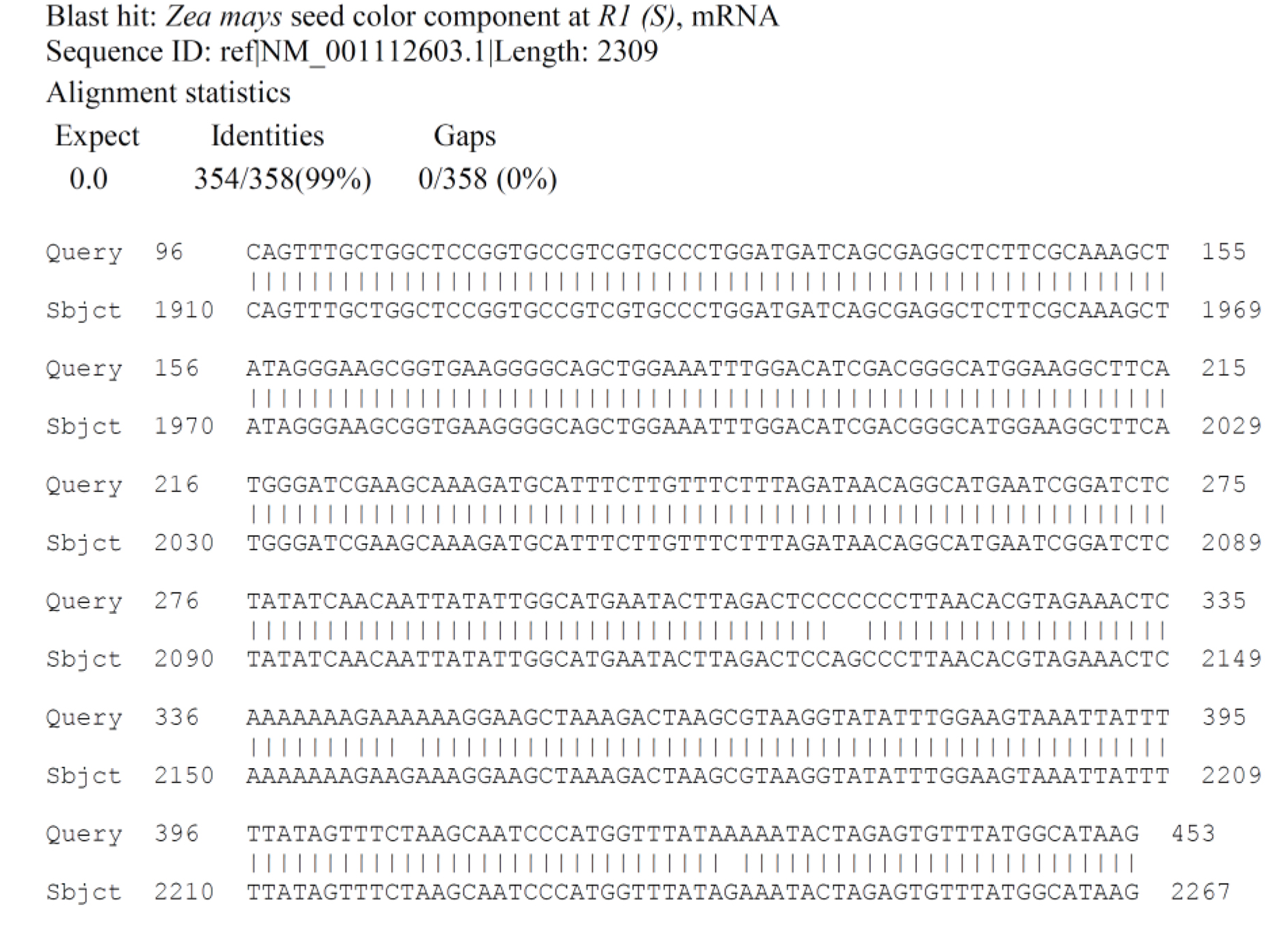

Supplement: S4 Fig — Alignment obtained by BLASTN program using as query the consensus sequence of 454 nucleotide at the 3' portion of r1 gene. (TIF) [file pone.0126521.s004.tif]
